# Web-Based Cognitive Behavioral Relapse Prevention Program With Tailored Feedback for People With Methamphetamine and Other Drug Use Problems: Development and Usability Study

**DOI:** 10.2196/mental.4875

**Published:** 2016-01-06

**Authors:** Ayumi Takano, Yuki Miyamoto, Norito Kawakami, Toshihiko Matsumoto

**Affiliations:** ^1^ Graduate School of Medicine Department of Psychiatric Nursing The University of Tokyo Tokyo Japan; ^2^ Graduate School of Medicine Department of Mental Health The University of Tokyo Tokyo Japan; ^3^ National Institute of Mental Health Department of Drug Dependence Research National Center of Neurology and Psychiatry Tokyo Japan

**Keywords:** web-based, drug dependence, relapse prevention, cognitive behavioral therapy, motivational interviewing, self-monitoring, Internet

## Abstract

**Background:**

Although drug abuse has been a serious public health concern, there have been problems with implementation of treatment for drug users in Japan because of poor accessibility to treatment, concerns about stigma and confidentiality, and costs. Therapeutic interventions using the Internet and computer technologies could improve this situation and provide more feasible and acceptable approaches.

**Objective:**

The objective of the study was to show how we developed a pilot version of a new Web-based cognitive behavioral relapse prevention program with tailored feedback to assist people with drug problems and assessed its acceptance and usability.

**Methods:**

We developed the pilot program based on existing face-to-face relapse prevention approaches using an open source Web application to build an e-learning website, including relapse prevention sessions with videos, exercises, a diary function, and self-monitoring. When users submitted exercise answers and their diary, researchers provided them with personalized feedback comments using motivational interviewing skills. People diagnosed with drug dependence were recruited in this pilot study from a psychiatric outpatient ward and nonprofit rehabilitation facilities and usability was evaluated using Internet questionnaires. Overall, website usability was assessed by the Web Usability Scale. The adequacy of procedures in the program, ease of use, helpfulness of content, and adverse effects, for example, drug craving, mental distress, were assessed by original structured questionnaires and descriptive form questions.

**Results:**

In total, 10 people participated in the study and completed the baseline assessment, 60% completed all relapse prevention sessions within the expected period. The time needed to complete one session was about 60 minutes and most of the participants took 2 days to complete the session. Overall website usability was good, with reasonable scores on subscales of the Web Usability Scale. The participants felt that the relapse prevention sessions were easy to use and helpful, but that the length of the videos was too long. The participant who until recently used drugs was satisfied with the self-monitoring, but others that had already maintained abstinence for more than a year felt this activity was unhelpful and were bored tracking and recording information on daily drug use. Feedback comments from researchers enhanced participants’ motivation and further insight into the disease. Serious adverse effects caused by the intervention were not observed. Some possible improvements to the program were suggested.

**Conclusions:**

The Web-based relapse prevention program was easy to use and acceptable to drug users in this study. This program will be helpful for drug users who do not receive behavioral therapy. After the pilot program is revised, further large-scale research is needed to assess its efficacy among drug users who have recently used drugs.

## Introduction

### Drug Use Problems and Treatments

Drug abuse is a serious public health problem all over the world. In the latest report, 243 million people, corresponding to 5.2% of the world population ages 15-64, have used an illicit drug at least once in the previous year [1]. In Japan, drug use in the community-based population has been much lower than that of other countries. Lifetime prevalence of drug use was estimated at 2.6% for any drug [2], 6.4% for nonmedical use of prescription drugs, and 1.5% for cannabis [3]. As for twelve-month prevalence, any drug use and drug dependence were reported as very close to zero [2,4]. However, there are high-risk groups with lifetime prevalence of any drug use estimated as 54.7% and 65.0% among HIV positive patients and men who have sex with men, respectively [5]. Lifetime prevalence of cannabis was reported as 24.7% among clubgoers [6]. These numbers for drug use prevalence were about 25 or more times higher than among the general population. The prevalence rate of drug use and drug dependence is considered underestimated because patients and high-risk people are unlikely to answer a nationwide survey. In fact, the numbers for people under arrest due to drug-related crimes and patients with drug dependence has remained stable or slightly increased [2,7].

The most prevalent drug has been methamphetamine in the treatment population, estimated at about 40% of patients who received any treatment in psychiatry with dependence or related disorders [8]. In recent years, health problems have arisen from rapidly increased use of new psychoactive substances (NPS), such as marijuana or stimulants containing synthetic cannabinoid and cathinone, especially among males in their 20s and 30s [8]. Prescription drug abuse has also increased, especially among females suffering from mental distress [8].

Many treatment interventions to prevent relapse have contributed to recovery from drug addiction. One of the most common and evidence-based approaches for several forms of drug addiction is behavioral therapy [9]. Behavioral therapies focus on various behavioral aspects and involve addressing drug users’ motivation to change, engage in treatments, handle triggers for drug cravings, acquire skills to resist drug use, replace activities using drug with constructive, and reward activities, and improve ways to handle problems and stress in various situations [10]. In previous meta-analysis, behavioral therapy demonstrated efficacy for abstinence with moderate effect size (d=0.45) and for treatment retention [11].

Despite evidence of effective and positive outcomes from behavioral therapies, treatment implementation remains problematic for various reasons. First, proper treatment is hampered by limited availability, rigid session times, inconvenient locations, and cost for service users [12,13]. Additionally, concerns about confidentiality and stigmatization further constrain drug users’ motivation to seek and engage in treatment [13]. Finally, the provision of frequent face-to-face validated interventions by well-trained therapists tends to create an economic and human-resource burden [14].

As for the treatment situation in Japan, there is also a gap between potential population-based treatment needs and available treatment services. Only about 16% of people received any psychiatric treatment among those with past alcohol/drug use disorders [15]. If they visit a psychiatrist, only 38.5% received specialized treatment for dependence, including cognitive behavioral therapy (CBT) [7]. This means that most drug users are not likely to visit a psychiatrist, nor would one receive specialized treatment even if treated by a psychiatrist. There are a few available community-based and evidence-based treatments because of a zero tolerance policy [16]. Many psychiatric hospitals have often only provided treatment for detoxification and medication in an acute stage without follow-up and psychosocial treatment [16,17]. Additionally, stigma and prejudice against drug use and drug users is also high among Japanese people including health care professionals [16]. As such, it is necessary to increase the availability and accessibility of evidence-based treatments that drug users can use without concerns about stigmatization and confidentiality.

### Intervention Using Computer and Internet Technology

Over the last two decades, interventions using computer technologies and the Internet have rapidly developed and adapted to various health problems, including for substance abuse, to address challenges in treatment implementation [12,14,18,19]. There are various types of Internet-based programs and most previous implementations were developed based on traditional face-to-face approaches and theories [20].

In Western countries, especially, many interventions have been developed to treat drug abuse. Enduring abstinence, engagement in treatment, actual help seeking, and cost effectiveness have been demonstrated by randomized controlled trials [13,14,21-28]. However, previous meta-analysis and systematic review studies revealed that the number of specific studies for drug abuse was fewer than for mental health problems, including alcohol and tobacco use problems [18,29]. Although there have been some Web-based or computerized intervention studies for cocaine or cannabis users, very few addressed users of various drugs including methamphetamine [19,30]. In addition, previous studies were heterogeneous in sample size, participant characteristics, applied technology type, presence and extent of therapist involvement, intervention length and session times, and condition of control group. As such, it has not been clear how to best implement interventions using a computer and Internet technologies for people with problems with various drugs including methamphetamine, especially in Asian countries. In Japan, while the availability of computer technologies and diffusion of Internet infrastructure has become widespread, technological treatments with validated methods remain underdeveloped.

The aims of this study are: (1) to describe the development of a new Web-based relapse prevention program with tailored feedback from a therapist using evidence-based cognitive behavioral approaches; and (2) to evaluate the acceptance and usability of the pilot program.

## Methods

### Development of the Pilot Version

#### Referenced Program

The new Web-based program was based on an existing evidence-based face-to-face relapse prevention program for people with drug use problems in Japan called the Serigaya Methamphetamine Relapse Prevention Program (SMARPP). SMARPP was developed in 2006 based on the Matrix Model as one of various effective behavioral therapies for outpatients with stimulant dependence [31-36]. The Matrix Model is a packaged cognitive behavioral relapse prevention program constructed with treatment elements based on other evidence-based approaches using detailed treatment manuals and demonstrated effectiveness for drug and alcohol reduction and risky sexual behaviors [33-36]. SMARPP inherits principles of the Matrix Model and aims at enhancing motivation for treatment and reducing drug use. The program is versatile and can be used for various drug problems. The program consists of a series of sessions based on educational components and practical relapse prevention exercises using a workbook and is done on a weekly basis [31]. SMARPP has been adapted for use to address different drug user needs and for various service providers, such as: the outpatient ward of psychiatric hospitals, the forensic psychiatric inpatient ward, the public mental health welfare center, nonprofit rehabilitation facilities, and probation offices. There are, however, challenges in implementation, particularly in community-based and outpatient settings.

As continuous drug-use monitoring is one of the important elements of treatment for drug addiction, participants of SMARRP check daily drug use and are encouraged to honestly convey their use to therapists and others. Urine tests and self-monitoring or self-monitoring only are also used depending on the institute and these results are only used to evaluate efficacy of the intervention and are kept confidential.

In previous studies, SMARPP participants showed more enduring abstinence, retention of outpatient treatment, and more frequent new enrollment in a self-help group than nonparticipants [37,38]. In addition, motivation for treatment and confidence dealing with drug cravings increased during intervention among inmates in a juvenile home and a prison that participated in the program [39,40].

#### Structure and Website Security

The new Web-based program was named e-SMARPP. The e-SMARPP website and content was developed by the first author primarily through the use of Moodle (version 2.6.1), which is an open-source Web application (app) to build e-learning websites [41]. Moodle has various modules to customize an original website written in PHP, which is a program language to make interactive Web pages, and is designed to support any device, including personal computers (PC), mobile phones, and tablet computers. An original domain name was obtained for the e-SMARPP website and access is encrypted through Secure Socket Layer technology. The e-SMARPP website is closed access. Each user is given an individual account that is created by only the first author using an email address and users create their nickname, identification, and password to log in.

#### e-Serigaya Methamphetamine Relapse Prevention Program Components

e-SMARPP is comprised of five parts: (1) a relapse prevention course, cognitive behavioral relapse prevention sessions (watching videos, submitting exercises, and a weekly diary on the website); (2) self-monitoring, calendar that displays drug use status by color; (3) information, downloadable PDF information and website links to drug addiction support services; (4) user guide, how to use the system, frequently asked questions, and contact form to researchers; and (5) a survey, questionnaires for baseline and post surveys. Users clicked radio buttons or input brief text when answering.

The main intervention contents are the relapse prevention course and the self-monitoring module. In the relapse prevention course, users are expected to complete each session over a week and in consecutive order. In this tentative study, four sessions were made for four weeks. Each session included a different number of videos and exercises and one weekly diary activity (Table 1). In order to explore what was a suitable number of videos and exercises in each session and length of videos, we provided a different number of videos and exercises sessions in the pilot version. Therefore, the volume of videos varied in each session. The total minutes of videos in each session ranged from approximately 23 to 66 minutes (Table 1).

**Table 1:** Content for relapse prevention course of e-SMARPP pilot version.

Session	Video	Minutes and seconds of video	Exercise	Weekly diary
1	Mental and physical consequences caused by drug use	8′ 39″	Think about your pros and cons for use/quitting drug use.	“How did you spend last week? How about drug use, emotions, events, etc?” “How will you spend next week? How about expected triggers, schedule, goals, etc?”
	What is dependence? Changes in the brain	12′ 24″	Define drug use situations: when, where, who, why, what, and emotion.	
	How to stop thinking about drugs	7′ 56″	Think about how to reduce cravings for drugs.	
2	Process of craving and drug use	6′ 38″		
	Triggers of craving	11′ 32″	Define your triggers.	
	Anchors keeping you from the drug	5′ 25″	Find your anchors.	
3	Process and stage of recovery	13′ 34″	Think of your signs of difficult times and barriers to recovery.	
	Safe lifestyle and signs of relapse	10′ 54″		
	How to plan a safe schedule	7′ 23″	Plan a safe holiday schedule without drugs.	
4	Novel psychoactive substances	11′ 13″		
	Prescription drug and over the counter drugs	14′ 23″		
	Alcohol	12′ 32″		
	How to quit or reduce alcohol	15′ 24″	Think about how to reduce or quit alcohol.	
	Cannabis	12′ 00″		

#### Video and Exercise Content

**Figure 1:**
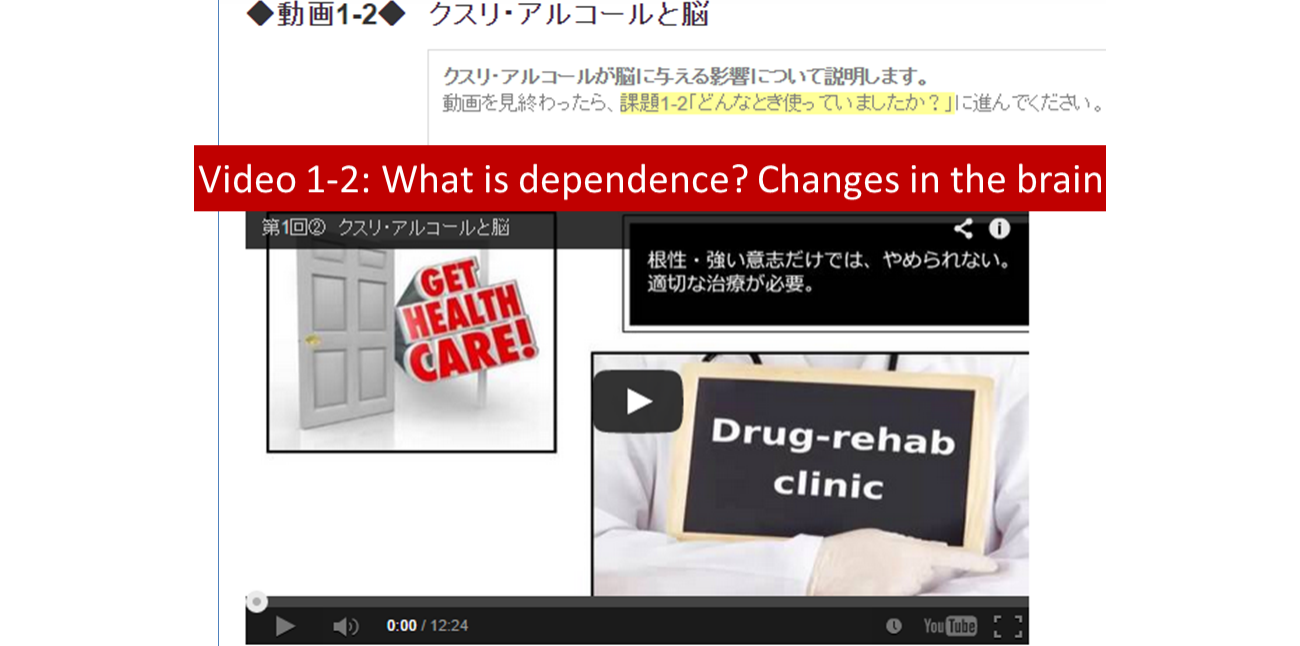
Video page screenshot.

**Figure 2:**
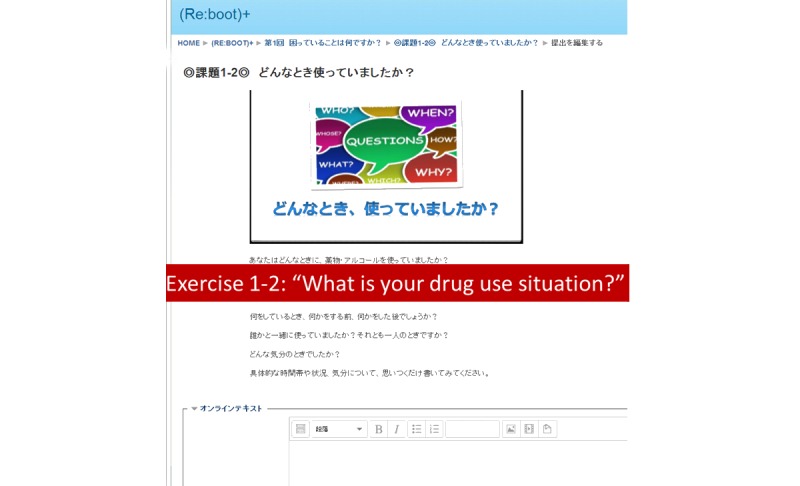
Exercise page screenshot.

Content for the videos and exercises were mostly taken from the SMARPP workbook. Although SMARPP has a considerable amount of content, we selected core content focused on encouraging drug users who have just started to seek treatment. These contents did not depend on the type of drug. We also added content from relevant books and websites if needed. Videos made in a YouTube format were embedded in each session (Figure 1 shows this). Narration and subtitles helped users understand the content. Exercises were based on the video content and users were expected to complete the exercises after watching the videos. Users wrote their own answers on an Internet text form and submitted the forms (Figure 2 shows this). As for the weekly diary activity, users were expected to write down their condition from the last week, current goals, and how they planned to spend time over the next week. Writing for the diary was also done on the Internet through the system. After submission of exercises and the weekly diary, users received tailored feedback from researchers.

**Figure 3:**
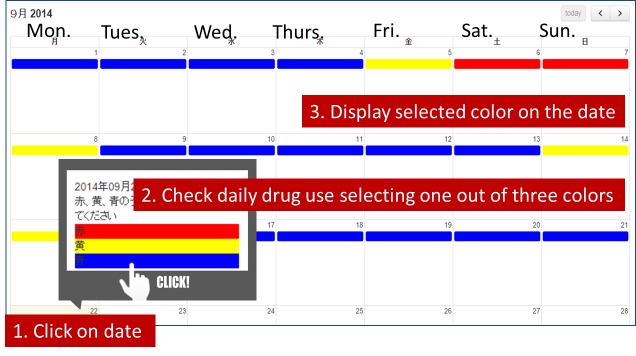
Self-monitoring calendar.

The self-monitoring calendar in e-SMARPP was newly developed, using a plug-in from Moodle, to provide a function similar to the self-monitoring process utilized in SMARPP. Participants clicked on a date in the calendar and selected one of three colors (red, yellow, or blue), with that color subsequently displayed on the date (Figure 3 shows this). The colors represent drug use: red reflecting abuse of the primary drug; yellow reflecting secondary abuse of other drugs and alcohol use, or alcohol use; and blue indicating no drug or alcohol use. Instructions and a legend for the colors were not displayed on the Web page to avoid concerns about confidentiality. At registration, users were given an explanation about the colors and how to use the calendar. This calendar only attempted to assess daily drug use without quantity or frequency-a-day. Because primary abuse of drugs were considered to vary and could not be adequately compared, we prioritized the development of a user-friendly program, and as such, did not provide several options for drug names and units and involve a complicated calculation system.

The self-monitoring calendar was also similar to the Timeline Followback (TLFB) method that retrospectively assesses drug use [42,43]. Although the TLFB method was developed to obtain self-reports on alcohol use with a paper-and-pencil approach, it has been extended to other behaviors and moreover Web-based versions have been developed with good reliability and usability [44-47]. During the intervention, participants were expected to check daily drug use and submit this at the weekly deadline (each Sunday).

#### Human Involvement and Automated Functions

Researchers qualified as health care professionals (registered nurse or medical doctor) and trained to support substance use disorders, provide personalized feedback comments within one or two days every time participants submitted exercises and the weekly diary. Feedback comments were based on motivational interview skills to enhance user motivation and to provide individual support. The researchers would send the users an email reminder on Monday if the users did not submit their exercises, diary, and self-monitoring calendar by each Sunday deadline.

e-SMARPP had some automated functions, including tracking progress for users, and a notification email for users when they received feedback, and for researchers when users submitted an exercise, diary, and questionnaire. In the notification emails, a related Web page link, for example, feedback comments page, is shown and users can access the Web page directly.

### Acceptance and Usability Study

#### Participants

We enrolled adults 20 years old and older who had been diagnosed in the past with substance use disorders, excluding alcohol, at the outpatient ward of the National Center of Neurology and Psychiatry (NCNP) and at nonprofit rehabilitation Drug Addiction Rehabilitation Center (DARC) institutes located in the Kanto region of Japan from March to April in 2014. DARC is operated by peer staff that have experienced drug dependence problems and are recovering at DARC. Additional inclusion criteria were: (1) those who could access the Internet via PC, mobile phone, and tablet, or tablet computer and exchange emails; (2) those without serious physical and psychiatric symptoms; and (3) with permission for participation in this study by psychiatrists at the NCNP or from DARC staff. Participants were given an explanation about this study with flyers provided by the researchers. At DARC, we asked DARC staff to refer DARC users to researchers and to participate in the study voluntarily. Because this study was a pilot and a first trial, we sought out a variety of comments from people dealing with drug problems at different stages of recovery. We thought opinions of people who had experience with drug use and recovery from drug dependence were of some help to developing an effective and user-friendly program. Therefore, we also invited DARC staff that had quit using drugs for more than a year to participate. In total, 12 people (the NCNP=3 and DARC=9) volunteered to participate.

#### Procedure

Applicants were given an explanation about the aims and procedures of this study. If they agreed to participate, they were registered at the e-SMARPP website by the first author and given an explanation about how to use e-SMARPP and participate in the tasks in this study. Then, they were asked to complete the baseline assessment on e-SMARPP and received a prepaid card for 1000 yen as a reward.

Participants participated in the program over four weeks. During each week, they were to go through a session and submit self-monitoring data on Sunday. If they did not complete the session and the self-monitoring by each deadline, researchers sent emails once or twice as a reminder. Information and a user guide were optional content. After the four weeks, participants completed a post survey.

The Ethics Committee of The University of Tokyo and the Ethics Committee of the NCNP approved this study. We received approval from the staff director of DARC before recruitment.

#### Measures

##### Baseline Assessment

Sociodemographic information was gathered including age (years), sex, marital status, cohabiter, educational history, employment status, and condition of Internet use (use days per week, hours per day, and main devices to access).

Information about history of drug use was also gathered. Primary drug of problems was assessed with the optional category of drug (methamphetamine, cannabis, NPS, prescription drug, organic solvent, cocaine, lysergic acid diethylamide: LSD, 3, 4- methylenedioxymethamphetamine: MDMA, heroine, over-the-counter drug, and other). If participants answered NPS as a primary drug, we also asked about its form (herb, liquid, and powder). Drug use in the past 28 days was collected using the self-monitoring calendar based on the TLFB method. In addition, we assessed multi-substance abuse (yes/no), onset age (years), first-abused drug as in the same category as the primary-abused drug, abstinence duration calculated from the day when they last used a drug, experience of past arrest (yes/no), past experience in a correctional facilities (yes/no), and self-reported psychiatric comorbidity with an option to select a diagnosis based on the International Classification of Diseases-10. Similarly, we evaluated history of treatment in several ways: duration of psychiatry outpatient ward, number of psychiatry hospitalization, specialized treatment for drug problems in the past (yes/no), and self-help group use (yes/no).

In order to assess severity of drug use problems in the past year, we used the Japanese version of the Drug Abuse Screening Test (DAST-20), which consists of 20 binary items [48,49]. Total score ranges from 0 to 20 and a high score represents a severe condition. The total score was classified into the following five levels: None (0), Low (1-5), Intermediate (6-10), Substantial (11-15), and Severe (16-20). Furthermore, the Kessler-6 (K6) scale consisting of six items measured on a 5-point scale was used to assess psychological distress [50,51]. Total score ranged from 0 to 24 and a high score indicates severe distress. The optimal cut-off point is considered 4/5 for a mood and anxiety disorder [52].

##### Acceptance Outcome

The compliance rate for the intervention and surveys among applicants was evaluated. The post survey asked participants about devices used and where they most frequently accessed e-SMARPP. Self-reported time needed to complete one exercise and one weekly diary and days needed to complete one session were assessed. We also asked about user experiences through four original questions: (1) “Do you think that number of session times was suitable?”, (2) “Do you think that the length of the videos was suitable?”, (3) “Do you think that exercises and the diary were difficult to answer?”, and (4) “Did you feel any harmful effects, for example, craving drugs or mental distress while using e-SMARPP?”

##### Usability Outcome

The Web Usability Scale was used to assess the overall usability of the e-SMARPP website. The Web Usability Scale consists of 21 items on a 5-point scale (1=disagree, 5=agree) and subscales: ease of use, ease in understanding structure, ease in reading, response speed, favorable, helpfulness, and credibility [53]. The average scores of each subscale were calculated and compared to each other. The Cronbach alpha coefficient in this study was .90, which meant the scale had good internal consistency. Next, to evaluate content usability in detail, the original 5-point scales (difficult, unhelpful, inadequate-easy, helpful, or adequate) were used as follows: degree of ease of use and helpfulness of content, degree of adequacy of feedback comments, and the most helpful/unhelpful content and their reasons. Finally, we gathered comments using description form questions to qualitatively evaluate content.

### Statistical Analysis

Descriptive statistics were used to examine the characteristics of participants and outcomes related to acceptance and usability of e-SMARPP. Data were analyzed using Microsoft Excel (Office 2010). The answers from the description form questions were summarized according to e-SMARPP content.

## Results

### Participant Characteristics

Of the 12 eligible applicants, 83% (10/12) completed the baseline assessment. There were two that were excluded because of bad health and an unknown reason. Sociodemographic characteristics of the ten participants are shown in Table 2. Most of the participants were male and recruited from DARC. About 70% (7/10) graduated high school or college and had a full-time or part-time job. Most accessed the Internet everyday primarily from a mobile phone (70%; 7/10) or PC (30%; 3/10).

**Table 2:** Participant demographic characteristics at baseline (n=10).

Demographic characteristics		n	%
**Sex**			
	Male	9	90
Age, mean (SD)		38.3	(5.6)
**Recruitment setting**			
	Outpatient ward of psychiatry	1	10
	Rehabilitation facility	9	90
**Marital status**			
	Married	3	30
	Never married	7	70
	Divorced/widowed	0	0
**Cohabiter**			
	Alone	3	30
	Partner/parent/child/other	7	70
**Years of education**			
	0-11 (< high school)	3	30
	12 (high school)	3	30
	13-15 (college)	4	40
	16+	0	0
**Employment**			
	Working (full-time)	4	40
	Working (part-time)	3	30
	Leave of absence	1	10
	Unemployed (employed in the past)	1	10
	Unemployed (never employed in the past)	0	0
	Student/homeworker	0	0
	Other	1	10
**Internet use outside of job** **(days per week)**			
	1/ 2/ 3/ 4	0	0
	5	1	10
	6	1	10
	7	8	70
**Internet use outside of job** **(hours per day)**			
	0-1	1	10
	1-2	5	50
	2-3	0	0
	3-4	2	20
	4-5	1	10
	5-6	0	0
	6-7	1	10
	7+	0	0
**Internet access devices** **(most used)**			
	PC	3	30
	Mobile phone	7	70
	Tablet computer	0	0

### History of Drug Use and Treatment

Table 3 shows history of drug use and treatment among participants. Primary drugs were methamphetamine (80%), cannabis (10%), and NPS (10%). The first-abused drugs were different and most participants used various drugs at the same time. Average age of onset for drug abuse was at 17.7 years old (SD 4.8, range: 12-29 years old). Most of the participants had maintained drug abstinence for more than a year and had been involved in a self-help group. Meanwhile, their severity of drug abuse and psychological distress tended to be relatively serious.

### Acceptance

#### Compliance Rate

Among ten participants who completed the baseline assessment, (60%) 6/10 completed all four sessions and (30%) 3/10 completed three or fewer sessions over the expected four weeks. Reasons for not completing sessions over the entire expected period were due to a full work schedule or reluctance. There was one participant (10%) that did not use e-SMARPP at all after the baseline assessment because of a recent family member death.

Of the ten participants who completed the baseline assessment, (80%) 8/10 responded to the post survey. However, there were missing variables because one participant (10%) did not complete a questionnaire.

#### User Experience

The device most commonly used to access e-SMARPP was a PC. Participants accessed most frequently from their home. The average minutes needed to complete one exercise and make diary entries were 9.5 (SD 5.6) and 9.3 (SD 5.7), respectively. This means that one session took about 43 to 86 minutes (average: about 60 minutes). Regarding days needed to complete one session, average days were 2.15 (SD 0.9) and median was 2.

Table 4 shows perceptions about e-SMARPP acceptance among the participants. More than half felt that the number of session times, one session per week, was suitable. However, many of them thought that the length of a video was too long. As for difficulty in responding to exercises and keeping a diary, more than half felt that this was basically easy. There were no participants that felt harmful effects while using e-SMARPP.

### Usability

#### Overall Usability of the e-Serigaya Methamphetamine Relapse Prevention Program Website

Table 5 shows the scores of each subscale of the Web Usability Scale used for assessing the overall usability of the e-SMARPP website. All average scores of the subscales were over 3 points. The highest score among the subscales was credibility and the worst was favorability.

**Table 3:** History of drug use and treatment among participants at baseline (n=10).

History of drug use and treatment		n	%
**Primary drug use problem** **(at baseline/ last drug problem)**			
	Methamphetamine	8	80
	Cannabis	1	10
	Novel psychoactive substances	1	10
	Other^a^	0	0
Multi-substance abuse	Yes	9	90
Onset age of drug abuse, mean (SD)	Range: 12-29	17.7	(4.8)
**First-abused drug**			
	Methamphetamine	1	10
	Cannabis	3	30
	Organic solvent	3	30
	Other	3	30
	Prescription/ designer drugs etc^b^	0	0
**Abstinence duration**			
	< 1 month	1	10
	1 month-1 year	0	0
	1 year-3 years	6	60
	> 3 years	3	30
Past arrest	Yes	7	70
Correction facility in the past	Yes	3	30
**Comorbidity** **(multiple answers)**			
	No	7	70
	Mood disorder	3	30
	Sleep disorder	1	10
Psychiatry outpatient	Yes	6	60
**Psychiatry outpatient length**			
	< 1 month	1	10
	< 6 month	2	20
	< 1 year	1	10
	> 1 year	6	60
Past psychiatry admission	Yes	4	40
Past CBT	Yes	3	30
Self-help group use	Yes	9	90
**Severity of drug abuse** ^c^ **DAST-20 scores, mean (SD)**			
	Range: 13-19	15.5	(2.0)
	None: 0/Low: 1-5/Intermediate: 6-10, n, (%)	0	(0)
	Substantial: 11-15, n, (%)	6	(60)
	Severe: 16-20, n, (%)	4	(40)
Psychological distress^d^, mean (SD)	Range: 1-16	7.3	(4.5)

^a^ Prescription, organic solvent, cocaine, LSD, heroin, MDMA, over-the-counter drugs and other.

^b^ Prescription, novel psychoactive substances, cocaine, LSD, heroin, MDMA, and over-the-counter drugs.

^c^ DAST-20, range: 0-20.

^d^ K6, range: 0-24.

**Table 4:** Perceptions about e-SMARPP acceptance (n=8).

Questions		n	%
**Number of session times**			
	Suitable	5	63
	Less is better	0	0
	More is better	0	0
	No opinion	3	38
**Length of a video**			
	Suitable	1	13
	Shorter is better	6	75
	Longer is better	0	0
	No opinion	1	13
**Difficulty in responding to exercise**			
	Difficult	0	0
	Slightly difficult	2	25
	No opinion	1	13
	Slightly easy	3	38
	Easy	2	25
**Difficulty diary entries**			
	Difficult	0	0
	Slightly difficult	2	25
	No opinion	1	13
	Slightly easy	2	25
	Easy	3	38
**Harmful effects**			
	No	7	88
	Yes	0	0
	Unknown	1	13

**Table 5:** Scores on Web Usability Scale to assess overall usability of e-SMARPP (n=7).

Subscales^a^	Mean	SD
Ease of use	3.48	0.92
Ease in understanding structure	3.43	0.68
Ease in reading	3.62	0.49
Response speed	3.43	0.95
Favorability	3.10	0.66
Helpfulness	3.71	0.60
Credibility	4.29	0.55

^a^ Each subscale consisted of 3 items on 5-point scale, 1=disagree, 5=agree.

#### Evaluation of Contents Usability

Tables 6 and 7 show data for the evaluation of usability of content and feedback comments. Videos were appreciated, however, some participants were not satisfied. Evaluation of exercise and diary was reasonable because some participants felt that these contents were helpful, but not easy to use. Evaluation of self-monitoring tended to be slightly negative. As for the evaluation of feedback comments from researchers, most of the participants thought that feedback comments were necessary and more than half of them felt that feedback comments were adequate and helpful. The most helpful content varied among participants and the least helpful content was self-monitoring.

**Table 6:** Evaluation of usability of e-SMARPP content (n=8).

Contents	Ease of use			Helpfulness		
	Easy, n (%)	Neutral/Unknown, n (%)	Difficult, n (%)	Helpful, n (%)	Neutral/Unknown, n (%)	Unhelpful, n (%)
Video	5 (63)	2 (25)	1 (13)	6 (75)	1 (13)	1 (13)
Exercise	4 (50)	2 (25)	2 (25)	7 (88)	0 (0)	1 (13)
Diary	5 (63)	2 (25)	1 (13)	7 (88)	0 (0)	1 (13)
Self-monitoring	2 (25)	4 (50)	2 (25)	2 (25)	2 (25)	4 (50)

**Table 7:** Evaluation of adequacy of feedback comments from therapists (n=8).

Feedback/comments	Adequacy			Helpfulness		
	Adequate, n (%)	Neutral, n (%)	Inadequate, n (%)	Helpful, n (%)	Neutral, n (%)	Unhelpful, n (%)
For exercise	5 (63)	3 (38)	0 (0)	5 (63)	2 (25)	1 (13)
For diary	5 (63)	3 (38)	0 (0)	5 (63)	2 (25)	1 (13)
Necessity, n (%)	7 (88)	1 (13)	0 (0)			

#### Qualitative Evaluation of Content

Both positive and negative comments for content were gathered using descriptive form questions. Overall, participants felt e-SMARPP was effective because cognitive behavioral approaches had different therapeutic elements from a self-help program or typical outpatient treatment, even if they had received some previous treatment and support.

Videos were well received, however, many participants felt the length was too long and hard to view on a small mobile phone screen,

The information was new and easy to understand. I learned some things that I didn’t know about. The videos were long, especially the one about people using drugs and having withdrawal symptoms. It was ambiguous that alcohol was included in drugs.

Male, an outpatient, DARC staffs and users

As for exercises and the weekly diary, many participants felt it valuable to have time to think about their problems and drug-use/nonuse schedule. They recognized that they were motivated and felt connected with supporters by feedback comments from the researchers. However, there was a comment that interaction with the researchers was limited. Some participants had some difficulty using the system and some Moodle functions over a mobile phone; for example, it was awkward to write long sentences. There was one participant that could not see feedback comments because he received garbled notification emails on the iPhone and could not access the Web page of the feedback comments directly. Although there were problems with compatibility in the character code on the iPhone email app, garbled characters were not found when he accessed the e-SMARPP website via a Web browser. Regarding the exercises,

I was able to think about my disease again. It was a good opportunity. I was motivated to see the videos because there were exercises. The feedback comments were good and encouraged me. It would be easier to respond if the questions were more focused and detailed.

Male, DARC staffs and users

Regarding the weekly diary,

I was able to review my life every week and confirm goals for the next week. It was valuable because it was the only part where I could freely write down some of my thoughts. Feedback comments gave me some calm and different ways of thinking.

Male, DARC staff and users

The comments about self-monitoring tended to be critical. There was one participant who just started to receive treatment that felt that it was helpful. On the other hand, participants who had received various support and were able to maintain drug abstinence for several years felt that it was not necessary to check their condition every day.

In addition, some participants mentioned technical and user interface difficulties, such as slow response speed or being likely to forget to check sometimes because the self-monitoring was put on a different Web page from the relapse prevention course. Some comments included,

It was not necessary for me because I have been able to maintain my drug abstinence. I did not feel that it was helpful because I checked the same color condition all at once. It was troublesome to input my daily condition. Batch input would be better if possible.

Male, DARC staffs

## Discussion

### Principal Results

We developed a pilot version of a Web-based cognitive behavioral relapse prevention program to assist people who want to address their drug problems. This e-SMARPP program was piloted and reasonably accepted, although the sample size of the study was small and participants’ ideas were a subset of all the ideas of the participants. The participants were satisfied with the relapse prevention sessions including videos, exercises, and diary activity; however, the self-monitoring was not favorably considered.

There were critical functional defects, although serious adverse events were not observed during the intervention. Further improvements are suggested.

### Development of the Pilot Version

When we developed the website and content, we tried to match the system to the needs of our target population. We discussed what a usable system would look like with drug users and therapists and incorporated their ideas into e-SMARPP. In one instance, we made videos with narrations and subtitles because some of those surveyed said they did not want to read Web pages with too much text and difficult Kanji characters. We decided to make content that was easy to understand and strived for favorable impressions by using videos. Additionally, we developed an accessible website and content that could be viewed via any device because mobile devices are very common in Japan for personal Internet use [54]. The Internet penetration rate is more than 90% among people age 13 to 59 in Japan and it has been increasing year by year [54]. Mobile phones have been more popular among young-middle age people, including drug users. In contrast, the Internet penetration rate is low among people with a low household income. Drug users who have withdrawal symptoms and mental disease comorbidity are considered to have difficulty with concentration and use of a Web-based program. Therefore, it is important to provide user-friendly content with a low cost. This will require further consideration in future revisions of the program.

We utilized an existing evidence-based face-to-face relapse prevention program to develop the new Web-based program based on previous studies [13,14,24,26]. The new Web-based program has key elements of a typical relapse prevention program and is promising in terms of efficacy for addressing drug use problems. In Japan, most face-to-face programs for drug users deal with problems of various drugs. Accordingly, e-SMARPP was developed with versatility to assist in handling common problems among drug users. We think e-SMARPP will be useful for researchers who want to know about programs for various substances, including methamphetamine.

Although a program with feedback from Web-therapists raises concern about scalability, we thought these functions to motivate users were necessary because treatment retention is very important for abstinence and recovery. Drug users mostly have ambivalent thoughts and it is essential to have motivational enhancement. A complete self-guided program requires more automated functions with complicated algorithms to support a personalized program. In addition, if we create a complete self-guided program and recruit drug users who do not receive any treatment and support by Internet or offline methods, we have to add a system to confirm the eligibility of people with drug problems and develop a different recruitment strategy. At this time, this was felt to be unrealistic for our study. We think e-SMARPP will be used as an extension of care or a partial replacement of standard treatment, similar to the work by Marsch et al [55], especially as at this point it might be difficult to recruit drug users who do not receive any treatment and support. Our program was similar to previous interventions in terms of intervention approach and adjunct treatment; however, there were some differences as our system was location independent and included human involvement by Web-therapists. The participants could use e-SMARPP anywhere, which was a more real world setting. They also obtained personalized feedback from researchers in a manner similar to real counseling. We plan to do a randomized controlled trial to assess the efficacy of our program, and then we hope to add a Web-recruitment system and recruit drug users who do not receive any treatment and support. If we can collaborate with primary care settings and community-based support, e-SMARPP will be widely used and helpful for those who are not receiving behavioral therapy.

### Acceptance and Usability Study

There are two thirds of the participants that completed all of the sessions and most felt that the frequency, one session a week, was suitable. There was one third that did not complete all the sessions because of other work commitments and low motivation. Participants took a total of approximately 60 minutes over two days to complete one session. This was not overly protracted, as the time needed to complete one session in this study was similar to times in other previous studies, where for example, in another program, one session took 45 minutes with a total of six sessions [14,24] and in another program, 60 minutes with a total of nine sessions [26]. In general, the session time of the Web-based interventions ranged from 20 to 60 minutes and tended to be shorter if the number of sessions was more frequent [12,19]. The number of sessions in an intervention study tends to be limited and session time tends to be longer than in a real setting because it is difficult to conduct frequent sessions over a long period, although intensive and long-term intervention is necessary to recover from drug dependence. For this reason, many participants felt the length of the videos were too long and also had difficulty watching them on a small mobile phone screen. In order to motivate busy and reluctant drug users to engage in the program, the videos should be shorter and include content focused on skills to avoid relapse, rather than educational content to provide knowledge about the dangers of drugs. Additionally, it is necessary to make improvements that facilitate a user-friendlier mobile phone interface. Measures to prevent drop out may also be necessary with a more suitable session time and improved content because the attrition rate of Web-based studies is likely to be higher than that of face-to-face interventions [56].

Most of the participants felt that the relapse prevention sessions were useful and helpful. They felt that there were new elements in the Web-based cognitive behavioral relapse prevention program even if they had gone through the 12-step program and/or psychiatric treatment. In contrast, except for one participant that was still currently using drugs, the majority of participants were not satisfied with the self-monitoring because it was boring for those who already had maintained a lengthy period of drug abstinence. The self-monitoring may be more important for drug users that currently or have recently used drugs. As such, the helpfulness and efficacy of the self-monitoring needs further study. While using the e-SMARPP, none of the participants felt an increased craving for drugs or added mental distress. This individual Web-based program may be suitable for drug users who are likely to be negatively influenced by others.

The results of the Web Usability Scale suggested that the overall usability of the website was reasonable. Most of the participants felt that the website and content were easy to use. However, one participant who used an iPhone received emails with unreadable characters because the characters were device-dependent. Functional defects, including garbled characters and the user interface for moving between Web pages, need to considered and revised.

### Future Directions

In accordance with the results of the acceptance and usability study, five key improvement items were identified: (1) simplify the content of the relapse prevention sessions; (2) improve usefulness of the self-monitoring; (3) prevent noncompletion of the intervention; (4) address functional defects; and (5) obtain real comments and ideas from participants and drug users through a focus group interview.

### Limitations

There were some limitations to this study. First, the generalizability of the results is limited due to the small sample size and some participants had previously received treatment and support, including CBT. Additionally, recruitment was done at only three settings. Second, usability and compliance in terms of Web-based programs depends on individual Internet literacy and computer skills [57,58], but we did not assess this. Finally, all outcome measures were self-reported, and not based on access logs, and as such, actual times needed to complete sessions may not be accurate.

### Conclusions

A new Web-based cognitive behavioral relapse prevention program for drug-abuse treatment, named e-SMARPP, was implemented and considered usable. Challenges were addressed and improvement points suggested. Although there are challenges to address in further development, this program and similar technology implementations are promising approaches for treatment in Japan, especially as drug-use stigma is strong in this society. After updates to the pilot version, further research is necessary to confirm its efficacy.

## Abbreviations

app: application
CBT: cognitive behavioral therapy
DARC: Drug Addiction Rehabilitation Center
DAST: Drug Abuse Screening Test
K6: Kessler-6 scale
NCNP: National Center of Neurology and Psychiatry
NPS: new psychoactive substances
PC: personal computers
SMARPP: Serigaya Methamphetamine Relapse Prevention Program
TLFB: Timeline Followback
